# Evolutionary and Expression Analysis of the Pig MAGE Gene Family

**DOI:** 10.3390/ani14142095

**Published:** 2024-07-17

**Authors:** Yu Zhang, Jian Tang, Yiwen Zheng, Wanshu Guo, Yuanyuan Guo, Minghang Chang, Hui Wang, Yanyan Li, Zhaoyue Chang, Yuan Xu, Zhipeng Wang

**Affiliations:** 1 College of Animal Science and Technology, Northeast Agricultural University, Harbin 150030, China; zhangyu22121@163.com (Y.Z.); tj15996815455@gmail.com (J.T.); zhengyiwen56@163.com (Y.Z.); guo10162022@163.com (W.G.); neau123456789@163.com (Y.G.); chang15632024@163.com (M.C.); wanghui19990709@163.com (H.W.); lyy5995992021@163.com (Y.L.); changzhaoyue2001@163.com (Z.C.); 2 Center for Bioinformatics, Northeast Agricultural University, Harbin 150030, China

**Keywords:** MAGE family, phylogenetic trees, pigs (*Sus scrofa*), gene expression

## Abstract

**Simple Summary:**

Most MAGE family genes have been identified on the mammalian genome and are located on the X chromosome. These genes are classified into type I and type II based on their sequence and function. *DreNDN* was the root of this evolutionary tree. In pigs, type I MAGE genes are predominantly expressed in the testis tissue, and type II MAGE genes are primarily expressed in the brain tissue. These findings are a valuable resource for acquiring insight into the evolution and expression of the MAGE family genes.

**Abstract:**

The melanoma-associated antigen (MAGE) family found in eukaryotes plays a crucial role in cell proliferation and differentiation, spermatogenesis, neural development, etc. This study explored the validation and evolution of MAGE genes in eukaryotic genomes and their distribution and expression patterns in pigs. In total, 249 MAGE genes were found on 13 eukaryotic species. In total, 33, 25, and 18 genes were located on human, mouse, and pig genomes, respectively. We found eight, four, and three tandemly duplicated gene clusters on the human, mouse, and pig genomes, respectively. The majority of MAGE genes in mammals are located on the X chromosome. According to the phylogenetic analysis, the MAGE family genes were classified into 11 subfamilies. The NDN gene in zebrafish (*DreNDN*) was the root of this evolutionary tree. In total, 10 and 11 MAGE genes on human and mouse genomes, respectively, exhibited a collinearity relationship with the MAGE genes on pig genomes. Taking the MAGE family genes in pigs, the MAGE subfamilies had similar gene structures, protein motifs, and biochemical attributes. Using the RNA-seq data of Duroc pigs and Rongchang pigs, we detected that the expression of type I MAGE genes was higher in reproductive tissues, but type II MAGE genes were predominantly expressed in the brain tissue. These findings are a valuable resource for gaining insight into the evolution and expression of the MAGE family genes.

## 1. Introduction

MAGE is a class of genes with a conserved sequence of the MAGE homologous structural domain (MHD) consisting of two tandem winged-helix (WH) motifs of 165–171 amino acids [1]. MAGEA1 was first identified in melanoma cells in 1991 [2]. Subsequent studies investigating MAGE homologous sequence genes have demonstrated that their homologs formed a multigene family in the mammalian genome. Moreover, a few MAGE homologous genes have also been found in zebrafish [3], chickens [4], Drosophila [5], and other non-mammals. Some studies have identified many MAGE genes on human and mouse genomes. Abera et al. [6] identified 34 and 31 MAGE genes on the human and mouse genomes, respectively. Based on RefSeq datasets and cDNA sequence searches, Zhao et al. (2011) [7] discovered 37 and 33 MAGE genes on the human and mouse genomes, respectively. These genes are classified into type I and type II MAGEs according to their sequence similarities and functional characteristics. Type I MAGE genes are encoded on the X chromosome and consist of three subfamilies: MAGEA, MAGEB, and MAGEC. The open reading frames of these genes are present in a single exon. The expression of type I MAGE genes is restricted to reproductive organs such as the testis, ovary, placenta, and uterus [8]. Additionally, type I MAGEs are also members of the cancer testis antigen (CTA) family. This gene family is abundantly expressed in various types of cancer cells. It also plays a major role in promoting cancer cell survival [9]. The type II MAGE gene family consists of seven major subfamilies: MAGED, MAGEE, MAGEF, MAGEG, MAGEH, MAGEL, and NDN. Type II MAGE genes are not confined to the X chromosome and are expressed in many tissues [10].

Because of the specific binding properties of the conserved MHD, individual MAGE proteins exhibit selective interactions with varying partners. This unique interaction pattern allows the MAGE family to regulate various biological processes, including metabolism, autophagy, DNA repair, cell cycle regulation, apoptosis, mRNA processing, and membrane protein recycling [11]. Numerous studies have reported that MAGE plays the critical roles in spermatogenesis [12], folliculogenesis [13], embryonic and germ cell development [14], cancer [15], and neuronal development [16]. For example, the results of MAGEB4 expressed during premeiotic stages suggest that MAGE genes are potentially involved in oocyte development [17].

The structure and biological functions of MAGE have been investigated. However, genome-wide validation and evolutionary analyses of MAGE genes in domesticated animals, such as sheep, horses, pigs, and cats, remain limited. Furthermore, structural analyses and expression studies of multi-tissues of MAGE genes in pigs are notably lacking. To gain deeper valuable insights into this gene family, the current study performed phylogenetic analysis of the MAGE gene family and examined the characteristics and expression of these genes in the porcine genome. The study findings are expected to strengthen our knowledge regarding the evolution, structure, and expression profile of porcine MAGE genes.

## 2. Materials and Methods

### 2.1. Validation of MAGE Family Members

The eukaryotic organisms included zebrafish (*Danio rerio*), chickens (*Gallus gallus*), pigs (*Sus scrofa*), cows (*Bos taurus*), sheep (*Ovis aries*), goats (*Capra hircus*), horses (*Equus caballus*), tigers (*Panthera tigris*), cats (*Felis catus*), dogs (*Canis familiaris*), mice (*Mus musculus*), rats (*Rattus norvegicus*), and humans (*Homo sapiens*) in this study. The genome and protein sequence files of 13 eukaryotes were downloaded from the ENSEMBL database (http://asia.ensembl.org/index.html, accessed on 10 January 2024) [18]. Using the hmmsearch tool, MAGE genes were searched (cutoff value: < 1 × 10^–20^). The CDD (https://www.ncbi.nlm.nih.gov/Structure/bwrpsb/bwrpsb.cgi, accessed on 12 January 2024) tool was employed for determining the conserved structural domains of protein sequences. The longest protein encoded by each MAGE gene was retained.

### 2.2. Phylogenetic Trees and Collinearity Analysis

Multiple sequence comparisons of the MAGE family of 13 species were performed using Mafft software (v7.487) [19] with the default parameter values. Phylogenetic trees were constructed and analyzed for their evolutionary relationships by using the Maximum Likelihood (ML) method through IQtree software (2.0) [20], with the bootstrap value set to 1000 and other parameters defaulted. The ITOL online tool (https://itol.embl.de/, accessed on 20 January 2024) [21] was used to draw the phylogenetic tree.

The genome and annotation files were used as input in MCScanX (https://www.rcac.purdue.edu/software/mcscanx, accessed on 22 January 2024) to map the actual locations of the MAGE genes on chromosomes [22]. TBtools was also used to analyze the MAGE genes’ collinearity relationships among the MAGE genes of pigs, humans, and mice [23], employing an e-value of 1 × 10^−3^.

### 2.3. Analysis of Protein Physicochemical Properties

The physicochemical properties of MAGE family proteins were predicted using the online tool ProtParam (https://web.expasy.org/protparam/, accessed on 8 February 2024) [24]. We determined the number of amino acids, molecular weight, isoelectric point, aliphatic index, and grand average of hydropathicity for each family member.

### 2.4. Motif and Gene Structure Analysis of MAGE Family Proteins

The conserved motifs of the MAGE family proteins were analyzed using MEME (http://meme-suite.org/, accessed on 10 February 2024) [25]. The motif length range was 10–60 amino acid residues. The exon and intron structures of the MAGE genes were obtained from the ENSEMBL gene annotation information. The diagrams of the MAGE gene structures were generated by GSDS (http://gsds.cbi.pku.edu.cn/, accessed on 10 February 2024) [26].

### 2.5. Expression Analysis of Pig MAGE Genes

The raw RNA sequencing (RNA-seq) data were downloaded from the NCBI Sequence Read Archive with the BioProject numbers PRJNA392949 [27] and PRJNA637678 [28]. It included 25 tissues (pancreas, breast, placenta, uterus, gall bladder, lung, testis, salivary gland, spinal cord, lymph, brain, urinary bladder, spleen, prostate, adrenal gland, nasopharynx, heart, ovary, thyroid, retina, fat, cell, gut, esophagus, and stomach) of adult Duroc pigs and adult Rongchang pigs.

All RNA-seq raw data were processed for quality control (QC) by using FastQC (v0.11.8). After QC, clean data were mapped and the genome indexed with STAR [29] to the pig genome (*Sus scrofa* 11.1). To determine the expression levels of genes across 25 tissues, the fragments per kilobase of the exon model per million mapped read (FPKM) values were calculated using the RSEM tool [30]. The tissue specificity index (τ) of the candidate gene was calculated, which is defined as follows:

$$\tau =\frac{\sum_{i=1}^{N}\left(1-x_{i}\right)}{N-1}$$

where *x_i_* represents the expression profile component normalized by the maximal component value, and *N* denotes the number of tissues. According to Yanai et al. (2005), genes with τ > 0.9 were considered tissue-specific genes [31].

## 3. Results

### 3.1. Validation Results of MAGE Family Members

We validated the members of the MAGE family in 13 species at the genome-wide level, including 2 representative species of non-mammalian (zebrafish and chickens) and 11 mammalian species (Table 1). In this study, 249 MAGE genes were found on the genomes of the 13 species. Few MAGE genes were observed on the genomes of the non-mammalian species. Only one MAGE gene was identified within the respective genomes of zebrafish and chickens. Numerous MAGE genes were observed on mammalian genomes, such as 18, 25, and 33 on the genomes of pigs, mice, and humans, respectively.

The MAGE gene family comprised 11 subfamilies. We unveiled the distinct distribution patterns of the MAGE subfamily genes across the genomes of different animals. The MAGEC subfamily genes were exclusively present in cattle and human genomes, and the MAGEG subfamily genes were unique to the chicken genome. The MAGEF subfamily genes were distributed across the genomes of nine mammalian species, except for rats and mice. The MAGEH subfamily genes were dispersed across the genomes of 10 mammalian species, except for dogs. Additionally, the NDN gene was dispersed across the genomes of 12 animal species, except for chickens.

### 3.2. Phylogenetic Analysis of the MAGE Family

**Figure 1:**
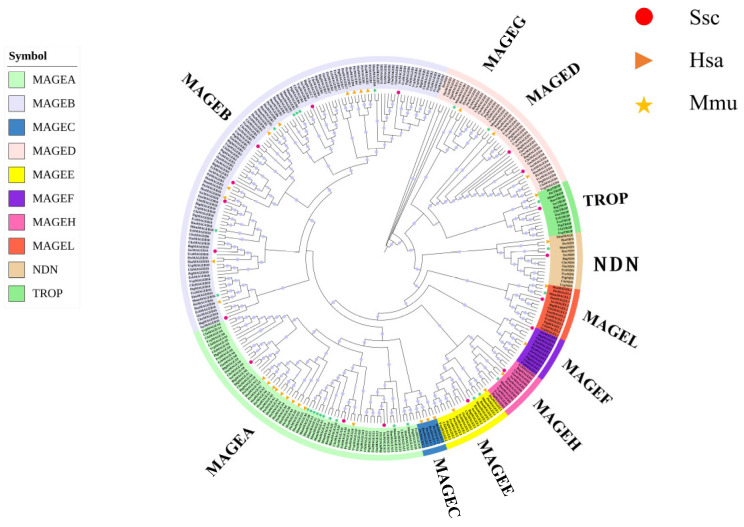
Phylogenetic tree of the MAGE family in 13 species. Gene identifier prefixes with species: Hsa stands for human, Mmu for mouse, and Ssc for pig.

The phylogenetic tree of the MAGE family, including all members of the 13 species, was constructed using the ML method (Figure 1). The results showed that the MAGE family genes formed 11 distinct branches, including the coalescence of MAGEA, MAGEB, and MAGEC in type I and the grouping of MAGED, MAGEE, MAGEF, MAGEG, MAGEH, MAGEL, NDN, and TROP in type II. The NDN gene in zebrafish (*DreNDN*) represented the evolutionary root of the generated phylogenetic tree. Significantly, the phylogenetic tree underscored the differences between type I and type II MAGE genes. The type I genes exhibited a more complex branching structure and a greater number of nodes, which indicated a more rapid expansion. Conversely, the branches in the type II MAGE gene clade formed were comparatively shorter, suggesting a slower rate of divergence compared to the type I MAGE genes.

### 3.3. Collinearity Analysis of the MAGE Family Genes between Pigs, Humans, and Mice

Approximately 90% MAGE family genes in humans, mice, and pigs are localized to the X chromosome. In humans, 30 of the 33 MAGE genes were mapped to ChrX and the others (*HsaMAGEF1*, *HsaMAGEL2*, and *HsaNDN*) on autosomes. Likewise, in mice, 22 of the 25 MAGE genes were localized on ChrX, except for *MmuMAGEB3*, *MmuMAGEL2*, and *MmuNDN*. Of the 18 porcine MAGE genes examined in pigs, 15 were located on the X chromosome, while *SscNDN* and *SscMAGEL2* were on Chr1 and *SscMAGEF1* on Chr13, respectively.

Two or more genes within a 200-kb region on a chromosome indicated the presence of tandemly duplicated genes in that region [32]. Tandemly duplicated genes preserve ancestral functions and contribute to the emergence of new functionalities. Three tandemly duplicated gene clusters were found on the pig genome. Of these gene clusters, two clusters were located on the X chromosome. One cluster was located between 22.08 and 22.18 Mb and contained the *SscMAGEB4*, *SscMAGEB5*, and *SscMAGEB18* genes. The other cluster was located between 26.04 and 26.08 Mb and contained the *SscMAGEB1* and *SscMAGEB3* genes. A tandemly duplicated gene cluster was identified on chromosome 1. It comprised *SscNDN* and *SscMAGEL2* within 142.41–142.46 Mb. Eight and four tandemly duplicated gene clusters were located on human and mouse genomes, respectively. In humans, seven tandemly clusters were located on the X chromosome, whereas one cluster was located on autosome 15. In mice, one tandemly duplicated gene cluster was located on chromosome 7, and the other clusters were found on the X chromosome.

**Figure 2:**
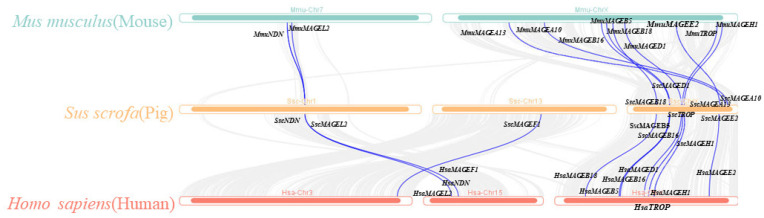
Collinearity analysis of MAGE family members.

To dissect the evolutionary relationships among the MAGE family members, we performed a cross-species collinearity analysis between pig and human and mouse genomes, respectively (Figure 2). In total, 10 and 11 MAGE genes were present on the human and mouse genomes, respectively, and exhibited a collinearity relationship with the pig MAGE genes. Because of the high degree of homology among collinear genes across species, they likely shared similar biological functions. The number of collinear genes was the highest on the X chromosome. Notably, the collinear genes of *SscMAGEA10* and *SscMAGEA13* were present on the mouse X chromosome but not on the human X chromosome. *SscMAGEF1* on chromosome 13 in pigs was only collinear with chromosome 3 in humans and exhibited no corresponding collinearity in mice. A tandemly duplicated gene cluster in pigs, comprising *SscMAGEL2* and *SscNDN* on chromosome 1, was collinear with tandemly repeated genes on chromosome 15 (*HsaMAGEL2* and *HsaNDN*) in humans and with tandemly repeated genes on chromosome 7 (MmuMAGEL2 and *MmuNDN*) in mice.

No collinearity relationships were observed between MAGE genes on human and mouse genomes and the pig genome, including *SscMAGEA8*, *SscMAGEB1*, *SscMAGEB3*, *SscMAGEB4*, *SscMAGEB10*, and *SscMAGED4*.

### 3.4. Analysis of the Protein Physicochemical Properties of Pig MAGE Genes

The physicochemical properties of the MAGE gene family in pigs, including chromosomal distribution, the number of amino acids, molecular weight (Da), theoretical isoelectric point (pI), aliphatic index, grand average of the hydropathicity index, and subcellular localization, were investigated (Table 2). Among the MAGE family proteins, *SscTROP* exhibited the most extensive amino acid sequence of 1258 amino acids, the minimal number of 219 amino acids in *SscMAGEH1*. *SscTROP* had the largest average molecular weight at 129,718.17 Da, whereas *SscMAGEH1* displayed the smallest molecular weight at 24,338.44 Da. Regarding isoelectric points, *SscMAGED1*, *SscMAGEA13*, *SscMAGEA10*, *SscMAGEA8*, *SscMAGEB16*, *SscMAGEE2*, and *SscMAGED4* displayed values less than 7, thereby indicating their acidity. In contrast, the remaining 11 members exhibited points more than 7, which indicated their alkaline characteristics. Notably, the *SscMAGEE2* protein displayed the highest aliphatic index and therefore had superior thermal stability, whereas the *SscTROP* and *SscMAGED4* proteins exhibited a lower aliphatic index, suggesting comparatively reduced thermal stability. Furthermore, the average coefficient of hydrophilicity across all 18 MAGE family members was negative, denoting that they were hydrophilic proteins.

### 3.5. Gene Structure and Motif Analysis of Porcine MAGE Family Genes

**Figure 3:**
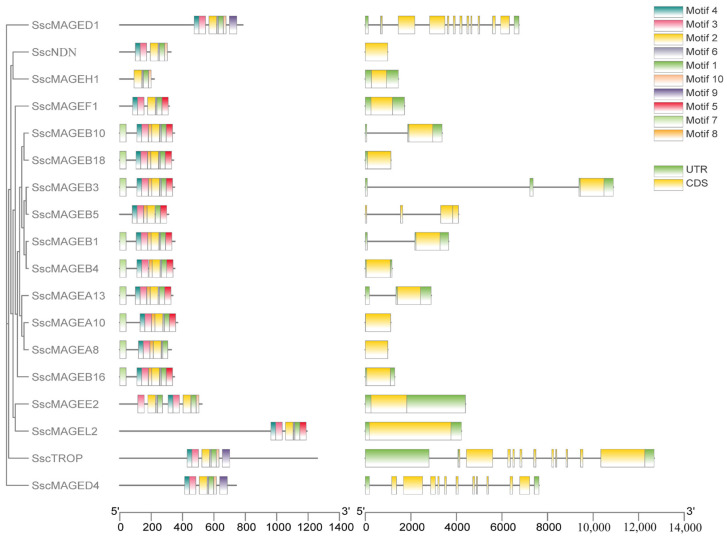
Gene structure and conserved motifs of porcine MAGE family members.

The gene structures and conserved motifs of the MAGE family members in pigs were analyzed using the online tools GSDS and MEME (Figure 3). The gene structure analysis recognized the substantial variation in exon numbers among MAGE family members, spanning 1 to 13. Most MAGE genes contained only a single exon, including *SscMAGEA8*, *SscMAGEA10*, *SscMAGEB4*, *SscMAGEB16*, *SscMAGEB18*, *SscMAGEE2*, *SscMAGEF1*, *SscMAGEH1*, *SscMAGEL2*, and *SscNDN*. The remaining MAGE genes harbored two to four exons, except the *SscMAGED* and *SscTROP* genes. These two genes had 13 and 12 exons, respectively. The motif analysis revealed that the MAGE family contains 10 motifs (see Figure 3). All MAGE proteins have motif1 and motif2, which probably denote the core functional domains. The *SscMAGEA* and *SscMAGEB* subfamilies contained identical motif1, motif2, motif3, motif4, and motif8, which implied that these two subfamilies may confer similar functions.

### 3.6. Expression Profile of MAGE Family Genes in Porcine Tissues

**Figure 4:**
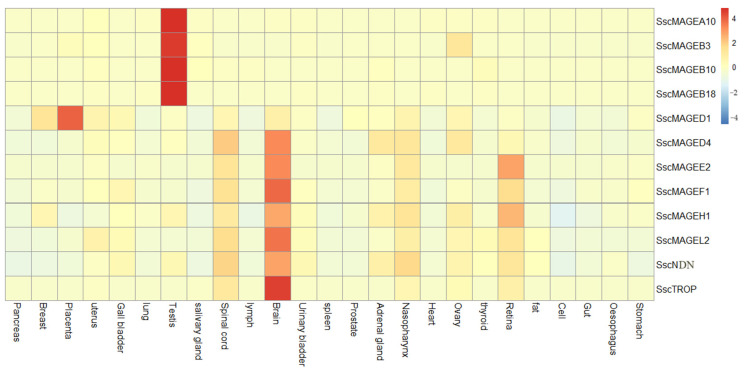
Expression heatmaps of MAGE family members in different tissues of Duroc pig.

To explore the expression profiles of the MAGE gene family in pigs, we gathered the RNA-seq data of 25 tissues of Duroc pigs and Rongchang pigs. The Duroc pig, a commercially significant breed, exhibits remarkable growth efficiency, with an average daily weight gain of 989 ± 125 g [33]. This breed is characterized by a moderate litter size, with an average of 9 to 10 piglets per farrowing [34]. In contrast, the Rongchang pig, a prominent indigenous Chinese breed, exhibits a comparatively lower growth rate [35]. However, it compensates with a high reproductive performance, yielding an average litter size of approximately 12 piglets [36]. For Duroc pigs, *SscMAGEA10*, *SscMAGEB3*, *SscMAGEB10*, and *SscMAGEB18* exhibited notably high expression within the testis tissues, yielding τ values of 0.9914, 0.9686, 0.9872, and 0.9969, respectively, and the *SscMAGEE2* gene was predominantly expression within brain tissues, yielding a tissue-specific expression index (τ) of 0.9060. For the expression levels of MAGE genes in various tissues of Duroc pigs, see Figure 4. For Rongchang pigs, *SscMAGEA10*, *SscMAGEB10*, and *SscMAGEB18* exhibited high expression within the testis tissues, yielding τ values of 0.9219, 0.9326, and 0.9309, respectively, and *SscMAGEE2* and *SscMAGEL2* were predominantly expressed within cerebrum tissues, yielding τ values of 0.9923 and 0.9273, respectively. In Rongchang pigs, the expression pattern of MAGE genes mirrors the expression observed in Duroc pigs. Specifically, type I MAGE gene families are predominantly expressed in reproductive tissues, whereas type II MAGE gene families are primarily expressed in brain and nerve-related tissues.

## 4. Discussion

In recent years, the continuous improvement and development of various sequencing technologies have led to the discovery of an increasing number of genes. This has also helped to identify new members of some gene families and understand the evolutionary history of the gene family and the biological functions of each gene in the family.

In this study, MAGE family members of 13 eukaryotic organisms were validated at the genome-wide level. MAGE is an ancient protein family that initially had a single copy gene in early eukaryotes [1]. Subsequent expansion in placental mammals resulted in the multi-gene family [5]. Studies have explained the mechanisms underlying the species-specific evolution of this gene family. We uncovered 249 MAGE genes on the genomes of 13 eukaryotic organisms and variable numbers of MAGE genes across the eukaryotes. The largest repertoire of MAGE genes existed in the human genome, whereas a single MAGE gene was identified in non-mammals. Katsura et al. [37] also reported a single MAGE gene on the zebrafish, frog, and chicken genomes, and the human genome harbored the greatest number of MAGE genes. This divergence may occur due to genomic duplication/deletion events coupled with different evolutionary selection pressures on different species. This implies that the MAGE family continues to expand during evolution, formatting a complete MAGE family in mammals. Gene duplication events are crucial for producing many vital developmental and regulatory genes found in extant species genomes [38,39,40].

According to the phylogenetic analysis, the *DreNDN* gene was the root of the MAGE family. Consistent with the findings of some previous studies, the *DreNDN* gene was the root of the phylogenetic tree of MAGE genes across 10 species, including *Bos taurus* (cattle), *Cavia porcellus* (tunicates), *Danio rerio* (zebrafish), *Gallus gallus* (chicken), *Homo sapiens* (human), *Macaca mulatta* (macaque), *Monodelphis domestica* (opossum), *Mus musculus* (mouse), *Ornithorhynchus anatinus* (platypus), and *Pan troglodytes* (chimpanzee) [32]. No found further scientific evidence was found supporting this hypothesis about *DreNDN* being the ancestral MAGE gene. However, some studies have suggested that the MAGED gene may be the ancestral gene of the MAGE family [41]. Taylor et al. (2008) proposed the MAGEG1 gene to be a founder of the MAGE gene family [42]. The ancestral MAGE gene remains debatable. More evidence is required to conclusively establish that a particular gene is the ancestor of the MAGE gene family.

The majority of MAGE family genes are localized to the X chromosome. This may be ascribed to the distinct gene recombination and replication characteristics of the X chromosome. Because of its pivotal role in reproductive functions, the X chromosome may have contributed to the pronounced concentration of MAGE genes on this chromosome [43].

The MAGE family encompasses type I and type II subdivisions, and differences exist between these two subdivisions. Type II genes include more subfamilies, although each type I subgroup has a greater total number of genes. Regarding origin, type II MAGE genes emerged earlier over the evolutionary history. Type I MAGE genes have undergone more rapid expansion of the gene family. Compared to type II MAGE genes, more type I MAGE genes are located on the X chromosome [44].

The genomic structures and phylogenetic relationships were similar among MAGEA and MAGEB subfamily members in pigs, which belonged to type I genes. Specifically, the MAGEA and MAGEB subfamily genes shared analogous exon numbers and motif compositions, which indicated that they had more similar functions in organisms. We investigated the MAGE gene expression pattern in different tissues of adult Duroc pigs. The expression of type I MAGE genes was higher in reproductive tissues. Clotman F et al. [45] investigated the MAGE gene expression pattern in mice, and the results showed that MAGEA and MAGEB were expressed at higher levels in the testis tissues. The knockout experiments of the MAGEA genes in mice demonstrated that these genes affect spermatogenesis [46].

Most type II genes, such as *SscMAGEE2*, *SscMAGED1*, *SscMAGEF1*, and *Ssc MAGEH1*, were predominantly expressed in the brain. Bertrand et al. [47] demonstrated that the MAGED subfamily genes were highly expressed in the neonatal mouse brain. The findings of Tsai [48] and Cheong [49] were in agreement with our findings that MAGED4 is highly expressed in the brain tissue. Furthermore, guided by brain-specific MAGEE2 expression in humans (GTEx Consortium 2015) [50], we demonstrated that MAGEE2 expression was special in the brains of adult pigs. Stone et al. [51] and Chang et al. [52] found that the expression of MAGEF1 and MAGEH1 is high in human brain tissues. NDN was ubiquitously expressed during the developmental stages of early neurons in mice [53,54]. What’s more, Chelly et al. [55] identified the MAGED genes associated with various monogenic X-linked neurodevelopmental disorders. MAGEL2 is critical for the pathogenesis of these disorders [56]. These results implied that type II MAGE genes have a substantial role in nervous system development and functioning.

## 5. Conclusions

The present study uncovered 249 MAGE genes across 13 eukaryotes and described their phylogeny tree. Most MAGE genes were located on the X chromosome. On the basis of pig multi-tissue expression profiles, we revealed that the expression of the MAGEA and MAGEB subfamilies were testis-specific and that of the type II MAGE gene was brain-specific. The results offer clues into the specialized regulatory modalities of MAGE genes in reproduction and neurobiology. This expression provides the foundation for elucidating the functional roles of MAGEs in pigs.

## Figures and Tables

**Table 1:** Distribution of MAGE family members in 13 species.

Type	Class	Fishs	Aves	Mammalia										
	Order	Osteicht-Hyes	Gallifor-Mes	Artiodactyla				Perissod-Actyla	Carnivora			Rodents		Primates
	Species	Zebrafish	Chicken	Pig	Cattle	Goat	Sheep	Horse	Tiger	Cat	Dog	Mouse	Rat	Human
I	MAGEA	0	0	3	5	4	4	3	3	3	5	10	4	10
I	MAGEB	0	0	7	9	10	9	8	9	8	8	7	8	10
I	MAGEC	0	0	0	1	0	0	0	0	0	0	0	0	3
II	MAGED	0	0	2	3	3	3	3	3	3	3	2	2	3
II	MAGEE	0	0	1	1	1	1	1	1	1	1	2	2	2
II	MAGEF	0	0	1	1	1	1	1	1	1	1	0	0	1
II	MAGEG	0	1	0	0	0	0	0	0	0	0	0	0	0
II	MAGEH	0	0	1	1	1	1	1	1	1	0	1	1	1
II	MAGEL	0	0	1	1	1	1	1	1	1	1	1	1	1
II	NDN	1	0	1	1	1	1	1	1	1	1	1	1	1
II	TROP	0	0	1	1	1	1	1	1	1	1	1	1	1
	Total	1	1	18	24	23	22	20	21	20	21	25	20	33

**Table 2:** Identification results of the physical and chemical properties related to the MAGE family in pigs.

Type	Gene Name	Chromosomal Localization (Mb)	Number of Amino Acid	Molecular Weight	Theoretical pI	Aliphatic Index	Grand Average of Hydropathicity	Subcellular Localization	
Type I	*SscMAGEA8*	chrX: 123.87	328	35,958.88	4.35	85.27	−0.404	plas: 21.5	
	*SscMAGEA10*	chrX: 123.40	368	40,252.98	4.56	69.7	−0.329	plas: 22.5	
	*SscMAGEA13*	chrX: 113.34	338	37,045.1	4.89	83.05	−0.264	cyto: 23.5	
	*SscMAGEB1*	chrX: 26.07–26.08	351	38,918.6	10.01	74.56	−0.64	nucl: 25.5	
	*SscMAGEB3*	chrX: 26.04–26.06	348	39,098.26	10.24	70.66	−0.577	nucl: 24	
	*SscMAGEB4*	chrX: 22.17	350	38,414.62	9.67	69.83	−0.678	nucl: 26	
	*SscMAGEB5*	chrX: 22.17–22.18	311	34,925.09	7.09	82.15	−0.424	nucl: 15.5	
	*SscMAGEB10*	chrX: 23.62	348	39,330.61	9.08	73.79	−0.63	cyto: 14	
	*SscMAGEB16*	chrX: 31.57–31.58	347	38,612	5.71	78.96	−0.382	cyto: 18.5	
	*SscMAGEB18*	chrX: 22.08	342	38,407.67	7.7	72.84	−0.611	nucl: 13	
Type II	*SscMAGED1*	chrX: 45.32–45.33	784	86,532.53	5.41	68.1	−0.554	cyto: 13.5	
	*SscMAGED4*	chrX: 0.03–0.04	742	81,377.52	6.52	64.66	−0.559	nucl: 23	
	*SscMAGEE2*	chrX: 60.71	523	60,076.19	4.84	87.5	−0.411	nucl: 12	
	*SscMAGEF1*	chr13: 122.60	315	36,006.57	9.33	83.05	−0.589	cyto: 24.5	
	*SscMAGEH1*	chr1: 48.08	219	24,338.44	9.36	65.48	−0.66	nucl: 25	
	*SscMAGEL2*	chr1: 142.45–142.46	1193	127,265.69	9.34	65.67	−0.461	nucl: 15	
	*SscTROP*	chrX: 47.76–47.77	1258	129,718.17	8.55	64.49	−0.188	nucl: 20	
	*SscNDN*	chrX: 142.41	325	36,381.87	9.05	81.11	−0.381	cyto: 18	

Note: plas, cyto, and nucl were plasma membrane, cytoplasmic, and nuclear, respectively.

## Data Availability

The expression data in this study were obtained from the Sequence Read Archive (SRA) database at the National Center for Biotechnology Information (NCBI) under accession numbers PRJNA392949 and PRJNA637678.
